# Bis(acetato-κ*O*)bis­(pyridine-2-aldoxime-κ^2^ *N*,*N*′)nickel(II)

**DOI:** 10.1107/S1600536812014377

**Published:** 2012-04-13

**Authors:** Youtao Si

**Affiliations:** a Universite de Europeenne de Bretagne, Universite de Bretagne Occidentale, CS 93837, 29238 Brest Cedex 3, France

## Abstract

In the mononuclear title compound, [Ni(CH_3_COO)_2_(C_6_H_6_N_2_O)_2_], the Ni^II^ atom is coordinated by two pyridine-2-aldoxime (PaoH) ligands and two acetate groups, with *cis* coordination for the pairs of identical ligands. While each acetate group binds to the Ni^II^ atom by one O atom, each PaoH chelates the Ni^II^ atom through two N atoms. The O atom on PaoH is not deprotonated and does not participate in bonding to the Ni^II^ atom. Thus, the Ni^II^ atom exhibits an octa­hedral environment. Intra­molecular O—H⋯O hydrogen-bonding inter­actions and inter­molecular C—H⋯O hydrogen-bonding inter­actions are present in the structure. Adjacent mol­ecules pack along [100] through van der Waals forces.

## Related literature

For [Ni(PaoH)_2_Cl_2_], see: Krause & Busch (1960 ▶); Miyasaka *et al.* (2004 ▶). 
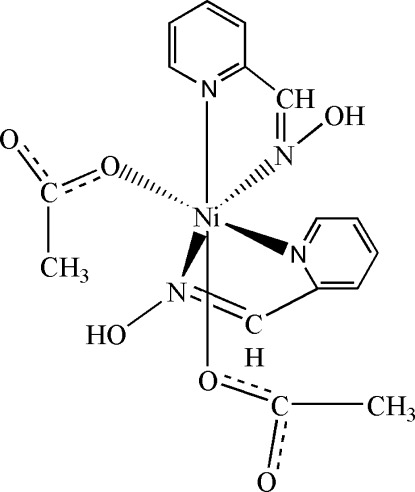


## Experimental

### 

#### Crystal data

[Ni(C_2_H_3_O_2_)_2_(C_6_H_6_N_2_O)_2_]
*M* *_r_* = 421.05
Monoclinic, 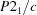

*a* = 8.649 (4) Å
*b* = 13.707 (7) Å
*c* = 17.775 (7) Å
β = 119.051 (17)°
*V* = 1842.1 (15) Å^3^
*Z* = 4
Mo *K*α radiation
μ = 1.09 mm^−1^
*T* = 293 K
0.20 × 0.20 × 0.20 mm

#### Data collection

Rigaku Mercury2 diffractometer
Absorption correction: multi-scan (*CrystalClear*; Rigaku, 2007) *T*_min_ = 0.809, *T*_max_ = 1.000
14112 measured reflections
4207 independent reflections
3298 reflections with *I* > 2σ(*I*)
*R*_int_ = 0.035

#### Refinement

*R*[*F* ^2^ > 2σ(*F* ^2^)] = 0.044
*wR*(*F* ^2^) = 0.113
*S* = 1.08
4207 reflections
244 parameters
H-atom parameters constrained
Δρ_max_ = 0.35 e Å^−3^
Δρ_min_ = −0.37 e Å^−3^

### 

Data collection: *CrystalClear* (Rigaku, 2007 ▶); cell refinement: *CrystalClear*; data reduction: *CrystalClear*; program(s) used to solve structure: *SHELXS97* (Sheldrick, 2008 ▶); program(s) used to refine structure: *SHELXL97* (Sheldrick, 2008 ▶); molecular graphics: *WinGX* (Farrugia, 1999 ▶); software used to prepare material for publication: *publCIF* (Westrip, 2010 ▶).

## Supplementary Material

Crystal structure: contains datablock(s) I, global. DOI: 10.1107/S1600536812014377/ru2032sup1.cif

Structure factors: contains datablock(s) I. DOI: 10.1107/S1600536812014377/ru2032Isup2.hkl

Supplementary material file. DOI: 10.1107/S1600536812014377/ru2032Isup3.cdx

Additional supplementary materials:  crystallographic information; 3D view; checkCIF report

## Figures and Tables

**Table 1:** Selected bond lengths (Å)

Ni1—O4	2.059 (2)
Ni1—O6	2.061 (2)
Ni1—N4	2.081 (2)
Ni1—N2	2.084 (2)
Ni1—N1	2.096 (2)
Ni1—N3	2.122 (2)

**Table 2:** Hydrogen-bond geometry (Å, °)

*D*—H⋯*A*	*D*—H	H⋯*A*	*D*⋯*A*	*D*—H⋯*A*
O1—H1*A*⋯O3	0.82	1.67	2.488 (4)	176
O2—H2*A*⋯O5	0.82	1.65	2.463 (3)	173
C3—H3*A*⋯O2^i^	0.93	2.43	3.307 (4)	157

Symmetry code: (i) 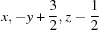.

**Fig. 1.**
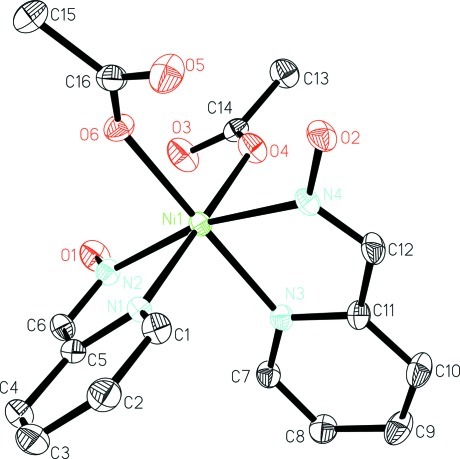
The molecular structure of the title compound, with atom labels and 20% probability displacement ellipsoids for all non-H atoms (H atoms omitted).

## supplementary crystallographic information

### Comment

Ni(PaoH)_2_Cl_2_ is a good coordination donor building for assembly with transition metal ions, like Mn^2+^. The reaction of Ni(PaoH)_2_Cl_2_ and Mn(OAc)_2_.4H_2_O afforded, unexpectedly, the title compound, in which AcO^-^ is substituted for Cl^-^.

*Cis* coordination fashion was found for the identical ligands. The average Ni—N bond length is 2.095 (18) Å, and the average Fe—O bond length is 2.060 (1) Å. The O atoms on PaoH remain protonated, and intramolecular O—H···O hydrogen-bonds are present, with the acceptor O atom from acetate.

### Experimental

Ni(PaoH)_2_Cl_2_ (0.19 g, 0.5 mmol) and Mn(OAc)_2_.4H_2_O (0.24 g, 1 mmol) were dissolved in DMF (1 mL) and MeOH (15 mL), then the mixture was stirred for 1 h, followed by filtration. After standing in air for 3 days, well shaped light purple crystals were obtained from the solution.

### Refinement

Hydrogen atoms were placed at idealized positions and allowed to ride on their parent atoms, with OH, CH and CH_3_ bonds set equal to 0.82, 0.93 and 0.96 Å, respectively. For H atoms of CH_3_, *U*_iso_(H) = 1.5*U*_eq_(C). For other H atoms, *U*_iso_(H) = 1.2*U*_eq_(C or O). The highest residual peak was located at 0.70 Å from H15C.

### Crystal data

**Table:**

[Ni(C_2_H_3_O_2_)_2_(C_6_H_6_N_2_O)_2_]	*F*(000) = 872
*M**_r_* = 421.05	*D*_x_ = 1.518 Mg m^−^^3^
Monoclinic, *P*2_1_/*c*	Mo *K*α radiation, λ = 0.71073 Å
Hall symbol: -P 2ybc	Cell parameters from 1810 reflections
*a* = 8.649 (4) Å	θ = 2.7–27.5°
*b* = 13.707 (7) Å	µ = 1.09 mm^−^^1^
*c* = 17.775 (7) Å	*T* = 293 K
β = 119.051 (17)°	Prism, light purple
*V* = 1842.1 (15) Å^3^	0.20 × 0.20 × 0.20 mm
*Z* = 4	

### Data collection

**Table:**

Rigaku Mercury2 diffractometer	4207 independent reflections
Radiation source: fine-focus sealed tube	3298 reflections with *I* > 2σ(*I*)
Graphite monochromator	*R*_int_ = 0.035
Detector resolution: 13.6612 pixels mm^-1^	θ_max_ = 27.5°, θ_min_ = 2.6°
CCD_Profile_fitting scans	*h* = −9→11
Absorption correction: multi-scan (*CrystalClear*; Rigaku, 2007)	*k* = −16→17
*T*_min_ = 0.809, *T*_max_ = 1.000	*l* = −23→23
14112 measured reflections	

### Refinement

**Table:**

Refinement on *F*^2^	Primary atom site location: structure-invariant direct methods
Least-squares matrix: full	Secondary atom site location: difference Fourier map
*R*[*F*^2^ > 2σ(*F*^2^)] = 0.044	Hydrogen site location: inferred from neighbouring sites
*wR*(*F*^2^) = 0.113	H-atom parameters constrained
*S* = 1.08	*w* = 1/[σ^2^(*F*_o_^2^) + (0.0526*P*)^2^ + 0.2764*P*] where *P* = (*F*_o_^2^ + 2*F*_c_^2^)/3
4207 reflections	(Δ/σ)_max_ = 0.001
244 parameters	Δρ_max_ = 0.35 e Å^−^^3^
0 restraints	Δρ_min_ = −0.37 e Å^−^^3^

### Special details

**Table:**

Geometry. All e.s.d.'s (except the e.s.d. in the dihedral angle between two l.s. planes) are estimated using the full covariance matrix. The cell e.s.d.'s are taken into account individually in the estimation of e.s.d.'s in distances, angles and torsion angles; correlations between e.s.d.'s in cell parameters are only used when they are defined by crystal symmetry. An approximate (isotropic) treatment of cell e.s.d.'s is used for estimating e.s.d.'s involving l.s. planes.
Refinement. Refinement of *F*^2^ against ALL reflections. The weighted *R*-factor *wR* and goodness of fit *S* are based on *F*^2^, conventional *R*-factors *R* are based on *F*, with *F* set to zero for negative *F*^2^. The threshold expression of *F*^2^ > σ(*F*^2^) is used only for calculating *R*-factors(gt) *etc*. and is not relevant to the choice of reflections for refinement. *R*-factors based on *F*^2^ are statistically about twice as large as those based on *F*, and *R*- factors based on ALL data will be even larger.

### Fractional atomic coordinates and isotropic or equivalent isotropic displacement parameters (Å^2^)

**Table:**

	*x*	*y*	*z*	*U*_iso_*/*U*_eq_	
Ni1	0.00171 (4)	0.80019 (2)	0.15100 (2)	0.03476 (13)	
O3	0.3495 (3)	0.88769 (19)	0.33957 (15)	0.0740 (7)	
O5	−0.4089 (3)	0.87646 (17)	0.10176 (16)	0.0686 (7)	
O6	−0.1317 (2)	0.92515 (14)	0.14997 (13)	0.0499 (5)	
O1	0.3372 (3)	0.92561 (17)	0.19987 (14)	0.0627 (6)	
H1A	0.3453	0.9146	0.2470	0.094*	
O4	0.1120 (3)	0.79396 (15)	0.28287 (12)	0.0531 (5)	
O2	−0.3346 (2)	0.70350 (14)	0.13957 (13)	0.0493 (5)	
H2A	−0.3671	0.7602	0.1271	0.074*	
N3	0.1359 (3)	0.67005 (16)	0.15232 (14)	0.0391 (5)	
N2	0.1841 (3)	0.88691 (16)	0.13709 (15)	0.0408 (5)	
N1	−0.1082 (3)	0.80866 (15)	0.01696 (14)	0.0370 (5)	
N4	−0.1728 (3)	0.69206 (16)	0.14512 (14)	0.0385 (5)	
C15	−0.3523 (4)	1.0447 (2)	0.1205 (2)	0.0618 (8)	
H15A	−0.4773	1.0458	0.1004	0.093*	
H15B	−0.3272	1.0807	0.0813	0.093*	
H15C	−0.2909	1.0738	0.1766	0.093*	
C3	−0.2199 (5)	0.8366 (3)	−0.1565 (2)	0.0603 (8)	
H3A	−0.2570	0.8451	−0.2147	0.072*	
C16	−0.2919 (4)	0.9406 (2)	0.12523 (19)	0.0482 (7)	
C7	0.2866 (3)	0.6588 (2)	0.14993 (18)	0.0443 (6)	
H7A	0.3472	0.7145	0.1490	0.053*	
C4	−0.0615 (4)	0.8770 (2)	−0.0945 (2)	0.0558 (8)	
H4A	0.0081	0.9139	−0.1104	0.067*	
C11	0.0493 (3)	0.5883 (2)	0.15438 (18)	0.0428 (6)	
C2	−0.3204 (4)	0.7842 (2)	−0.1307 (2)	0.0547 (8)	
H2B	−0.4277	0.7574	−0.1711	0.066*	
C14	0.2413 (4)	0.8323 (2)	0.34460 (18)	0.0436 (6)	
C1	−0.2604 (3)	0.7717 (2)	−0.04356 (18)	0.0450 (6)	
H1B	−0.3294	0.7358	−0.0266	0.054*	
C13	0.2694 (4)	0.8122 (2)	0.4337 (2)	0.0619 (9)	
H13A	0.1793	0.7685	0.4302	0.093*	
H13B	0.2633	0.8723	0.4599	0.093*	
H13C	0.3835	0.7829	0.4679	0.093*	
C5	−0.0093 (4)	0.86133 (19)	−0.00854 (18)	0.0413 (6)	
C12	−0.1192 (4)	0.6044 (2)	0.15257 (19)	0.0478 (7)	
H12A	−0.1846	0.5528	0.1567	0.057*	
C6	0.1540 (4)	0.9011 (2)	0.06049 (19)	0.0463 (7)	
H6A	0.2330	0.9355	0.0491	0.056*	
C9	0.2713 (4)	0.4870 (2)	0.1523 (2)	0.0652 (9)	
H9A	0.3170	0.4256	0.1522	0.078*	
C8	0.3571 (4)	0.5693 (2)	0.1487 (2)	0.0549 (8)	
H8A	0.4611	0.5648	0.1456	0.066*	
C10	0.1161 (4)	0.4964 (2)	0.1560 (2)	0.0607 (9)	
H10A	0.0572	0.4413	0.1595	0.073*	

### Atomic displacement parameters (Å^2^)

**Table:**

	*U*^11^	*U*^22^	*U*^33^	*U*^12^	*U*^13^	*U*^23^
Ni1	0.03189 (19)	0.0365 (2)	0.0371 (2)	−0.00362 (13)	0.01775 (15)	0.00059 (14)
O3	0.0659 (14)	0.0939 (19)	0.0554 (14)	−0.0353 (14)	0.0241 (11)	−0.0081 (13)
O5	0.0434 (12)	0.0570 (14)	0.1006 (19)	0.0026 (10)	0.0311 (12)	0.0034 (13)
O6	0.0412 (10)	0.0474 (11)	0.0631 (13)	0.0031 (8)	0.0270 (9)	−0.0046 (10)
O1	0.0501 (12)	0.0747 (15)	0.0596 (14)	−0.0285 (11)	0.0237 (10)	−0.0104 (11)
O4	0.0481 (11)	0.0691 (14)	0.0347 (11)	−0.0148 (10)	0.0142 (9)	−0.0021 (9)
O2	0.0356 (10)	0.0568 (13)	0.0626 (13)	−0.0030 (8)	0.0294 (9)	0.0071 (10)
N3	0.0350 (11)	0.0385 (12)	0.0424 (13)	−0.0019 (9)	0.0177 (9)	0.0026 (10)
N2	0.0339 (11)	0.0389 (12)	0.0492 (14)	−0.0081 (9)	0.0197 (10)	−0.0029 (10)
N1	0.0394 (11)	0.0374 (12)	0.0374 (12)	−0.0007 (9)	0.0212 (9)	0.0016 (9)
N4	0.0326 (11)	0.0460 (13)	0.0390 (12)	−0.0071 (9)	0.0190 (9)	0.0030 (10)
C15	0.0566 (18)	0.0544 (19)	0.069 (2)	0.0126 (15)	0.0265 (16)	−0.0026 (16)
C3	0.078 (2)	0.064 (2)	0.0404 (17)	0.0078 (17)	0.0296 (16)	0.0033 (15)
C16	0.0484 (16)	0.0555 (18)	0.0450 (16)	0.0083 (14)	0.0262 (13)	0.0021 (13)
C7	0.0359 (13)	0.0438 (15)	0.0555 (18)	0.0003 (12)	0.0239 (12)	0.0057 (13)
C4	0.074 (2)	0.0536 (19)	0.0545 (19)	0.0053 (15)	0.0422 (17)	0.0089 (15)
C11	0.0411 (14)	0.0374 (14)	0.0460 (16)	−0.0049 (11)	0.0181 (12)	0.0027 (12)
C2	0.0577 (18)	0.0531 (18)	0.0430 (17)	0.0019 (14)	0.0163 (14)	−0.0033 (14)
C14	0.0427 (14)	0.0414 (15)	0.0425 (16)	0.0054 (12)	0.0174 (12)	−0.0025 (12)
C1	0.0414 (14)	0.0475 (16)	0.0422 (16)	−0.0017 (12)	0.0173 (12)	0.0017 (13)
C13	0.064 (2)	0.073 (2)	0.0407 (17)	−0.0059 (16)	0.0194 (15)	−0.0016 (15)
C5	0.0504 (15)	0.0373 (14)	0.0450 (16)	0.0023 (12)	0.0301 (13)	0.0005 (12)
C12	0.0435 (15)	0.0425 (16)	0.0609 (19)	−0.0096 (12)	0.0280 (13)	0.0055 (13)
C6	0.0483 (15)	0.0457 (16)	0.0550 (18)	−0.0081 (12)	0.0331 (14)	0.0019 (13)
C9	0.0530 (18)	0.0430 (17)	0.093 (3)	0.0070 (14)	0.0307 (18)	−0.0010 (17)
C8	0.0417 (15)	0.0550 (18)	0.071 (2)	0.0072 (13)	0.0297 (15)	0.0049 (16)
C10	0.0506 (17)	0.0384 (16)	0.089 (3)	−0.0057 (13)	0.0307 (17)	−0.0003 (16)

### Geometric parameters (Å, º)

**Table:**

Ni1—O4	2.059 (2)	C3—C2	1.367 (5)
Ni1—O6	2.061 (2)	C3—C4	1.390 (5)
Ni1—N4	2.081 (2)	C3—H3A	0.9300
Ni1—N2	2.084 (2)	C7—C8	1.375 (4)
Ni1—N1	2.096 (2)	C7—H7A	0.9300
Ni1—N3	2.122 (2)	C4—C5	1.383 (4)
O3—C14	1.241 (3)	C4—H4A	0.9300
O5—C16	1.249 (4)	C11—C10	1.380 (4)
O6—C16	1.252 (3)	C11—C12	1.459 (4)
O1—N2	1.358 (3)	C2—C1	1.384 (4)
O1—H1A	0.8200	C2—H2B	0.9300
O4—C14	1.241 (3)	C14—C13	1.507 (4)
O2—N4	1.364 (3)	C1—H1B	0.9300
O2—H2A	0.8200	C13—H13A	0.9600
N3—C7	1.333 (3)	C13—H13B	0.9600
N3—C11	1.358 (3)	C13—H13C	0.9600
N2—C6	1.271 (3)	C5—C6	1.454 (4)
N1—C1	1.331 (3)	C12—H12A	0.9300
N1—C5	1.355 (3)	C6—H6A	0.9300
N4—C12	1.272 (3)	C9—C8	1.368 (4)
C15—C16	1.507 (4)	C9—C10	1.381 (4)
C15—H15A	0.9600	C9—H9A	0.9300
C15—H15B	0.9600	C8—H8A	0.9300
C15—H15C	0.9600	C10—H10A	0.9300
			
O4—Ni1—O6	89.56 (9)	N3—C7—C8	123.4 (3)
O4—Ni1—N4	87.21 (8)	N3—C7—H7A	118.3
O6—Ni1—N4	101.66 (9)	C8—C7—H7A	118.3
O4—Ni1—N2	101.53 (8)	C5—C4—C3	118.8 (3)
O6—Ni1—N2	88.73 (9)	C5—C4—H4A	120.6
N4—Ni1—N2	166.55 (9)	C3—C4—H4A	120.6
O4—Ni1—N1	179.06 (8)	N3—C11—C10	121.5 (3)
O6—Ni1—N1	90.05 (8)	N3—C11—C12	115.7 (2)
N4—Ni1—N1	93.71 (8)	C10—C11—C12	122.8 (3)
N2—Ni1—N1	77.60 (9)	C3—C2—C1	119.1 (3)
O4—Ni1—N3	90.23 (9)	C3—C2—H2B	120.4
O6—Ni1—N3	179.02 (8)	C1—C2—H2B	120.4
N4—Ni1—N3	77.37 (9)	O4—C14—O3	125.7 (3)
N2—Ni1—N3	92.24 (9)	O4—C14—C13	117.9 (3)
N1—Ni1—N3	90.17 (8)	O3—C14—C13	116.4 (3)
C16—O6—Ni1	131.6 (2)	N1—C1—C2	122.9 (3)
N2—O1—H1A	109.5	N1—C1—H1B	118.5
C14—O4—Ni1	135.2 (2)	C2—C1—H1B	118.5
N4—O2—H2A	109.5	C14—C13—H13A	109.5
C7—N3—C11	117.8 (2)	C14—C13—H13B	109.5
C7—N3—Ni1	129.37 (19)	H13A—C13—H13B	109.5
C11—N3—Ni1	112.80 (17)	C14—C13—H13C	109.5
C6—N2—O1	115.6 (2)	H13A—C13—H13C	109.5
C6—N2—Ni1	116.18 (18)	H13B—C13—H13C	109.5
O1—N2—Ni1	128.13 (18)	N1—C5—C4	122.1 (3)
C1—N1—C5	118.0 (2)	N1—C5—C6	115.5 (2)
C1—N1—Ni1	128.49 (18)	C4—C5—C6	122.4 (3)
C5—N1—Ni1	113.46 (17)	N4—C12—C11	117.0 (2)
C12—N4—O2	115.2 (2)	N4—C12—H12A	121.5
C12—N4—Ni1	116.72 (18)	C11—C12—H12A	121.5
O2—N4—Ni1	127.99 (16)	N2—C6—C5	117.2 (2)
C16—C15—H15A	109.5	N2—C6—H6A	121.4
C16—C15—H15B	109.5	C5—C6—H6A	121.4
H15A—C15—H15B	109.5	C8—C9—C10	119.1 (3)
C16—C15—H15C	109.5	C8—C9—H9A	120.4
H15A—C15—H15C	109.5	C10—C9—H9A	120.4
H15B—C15—H15C	109.5	C9—C8—C7	118.7 (3)
C2—C3—C4	119.0 (3)	C9—C8—H8A	120.6
C2—C3—H3A	120.5	C7—C8—H8A	120.6
C4—C3—H3A	120.5	C11—C10—C9	119.4 (3)
O5—C16—O6	125.2 (3)	C11—C10—H10A	120.3
O5—C16—C15	116.4 (3)	C9—C10—H10A	120.3
O6—C16—C15	118.3 (3)		
			
O4—Ni1—O6—C16	−108.2 (3)	N2—Ni1—N4—C12	46.2 (5)
N4—Ni1—O6—C16	−21.1 (3)	N1—Ni1—N4—C12	95.3 (2)
N2—Ni1—O6—C16	150.2 (3)	N3—Ni1—N4—C12	6.0 (2)
N1—Ni1—O6—C16	72.6 (3)	O4—Ni1—N4—O2	90.6 (2)
N3—Ni1—O6—C16	−30 (5)	O6—Ni1—N4—O2	1.7 (2)
O6—Ni1—O4—C14	−83.6 (3)	N2—Ni1—N4—O2	−138.3 (3)
N4—Ni1—O4—C14	174.7 (3)	N1—Ni1—N4—O2	−89.1 (2)
N2—Ni1—O4—C14	5.0 (3)	N3—Ni1—N4—O2	−178.5 (2)
N1—Ni1—O4—C14	−18 (5)	Ni1—O6—C16—O5	7.5 (5)
N3—Ni1—O4—C14	97.3 (3)	Ni1—O6—C16—C15	−170.6 (2)
O4—Ni1—N3—C7	−98.1 (2)	C11—N3—C7—C8	0.6 (4)
O6—Ni1—N3—C7	−176 (100)	Ni1—N3—C7—C8	−178.1 (2)
N4—Ni1—N3—C7	174.8 (3)	C2—C3—C4—C5	1.1 (5)
N2—Ni1—N3—C7	3.4 (2)	C7—N3—C11—C10	1.5 (4)
N1—Ni1—N3—C7	81.0 (2)	Ni1—N3—C11—C10	−179.7 (2)
O4—Ni1—N3—C11	83.15 (19)	C7—N3—C11—C12	−177.0 (2)
O6—Ni1—N3—C11	5 (5)	Ni1—N3—C11—C12	1.8 (3)
N4—Ni1—N3—C11	−3.93 (17)	C4—C3—C2—C1	−1.0 (5)
N2—Ni1—N3—C11	−175.29 (19)	Ni1—O4—C14—O3	−2.7 (5)
N1—Ni1—N3—C11	−97.69 (19)	Ni1—O4—C14—C13	176.1 (2)
O4—Ni1—N2—C6	177.5 (2)	C5—N1—C1—C2	0.4 (4)
O6—Ni1—N2—C6	−93.2 (2)	Ni1—N1—C1—C2	178.6 (2)
N4—Ni1—N2—C6	47.8 (5)	C3—C2—C1—N1	0.2 (5)
N1—Ni1—N2—C6	−2.9 (2)	C1—N1—C5—C4	−0.2 (4)
N3—Ni1—N2—C6	86.8 (2)	Ni1—N1—C5—C4	−178.6 (2)
O4—Ni1—N2—O1	1.8 (2)	C1—N1—C5—C6	179.1 (2)
O6—Ni1—N2—O1	91.1 (2)	Ni1—N1—C5—C6	0.6 (3)
N4—Ni1—N2—O1	−128.0 (4)	C3—C4—C5—N1	−0.6 (4)
N1—Ni1—N2—O1	−178.6 (2)	C3—C4—C5—C6	−179.8 (3)
N3—Ni1—N2—O1	−88.9 (2)	O2—N4—C12—C11	177.0 (2)
O4—Ni1—N1—C1	−154 (5)	Ni1—N4—C12—C11	−6.9 (3)
O6—Ni1—N1—C1	−88.5 (2)	N3—C11—C12—N4	3.3 (4)
N4—Ni1—N1—C1	13.2 (2)	C10—C11—C12—N4	−175.2 (3)
N2—Ni1—N1—C1	−177.2 (2)	O1—N2—C6—C5	−179.6 (2)
N3—Ni1—N1—C1	90.6 (2)	Ni1—N2—C6—C5	4.1 (3)
O4—Ni1—N1—C5	24 (5)	N1—C5—C6—N2	−3.2 (4)
O6—Ni1—N1—C5	89.74 (18)	C4—C5—C6—N2	176.1 (3)
N4—Ni1—N1—C5	−168.57 (18)	C10—C9—C8—C7	0.7 (5)
N2—Ni1—N1—C5	1.04 (17)	N3—C7—C8—C9	−1.7 (5)
N3—Ni1—N1—C5	−91.21 (18)	N3—C11—C10—C9	−2.3 (5)
O4—Ni1—N4—C12	−84.9 (2)	C12—C11—C10—C9	176.1 (3)
O6—Ni1—N4—C12	−173.8 (2)	C8—C9—C10—C11	1.2 (5)

### Hydrogen-bond geometry (Å, º)

**Table:**

*D*—H···*A*	*D*—H	H···*A*	*D*···*A*	*D*—H···*A*
O1—H1*A*···O3	0.82	1.67	2.488 (4)	176
O2—H2*A*···O5	0.82	1.65	2.463 (3)	173
C3—H3*A*···O2^i^	0.93	2.43	3.307 (4)	157

Symmetry code: (i) *x*, −*y*+3/2, *z*−1/2.
